# Design and Synthesis of ESIPT-Based Imidazole Derivatives for Cell Imaging

**DOI:** 10.1021/acsomega.3c09822

**Published:** 2024-05-27

**Authors:** Sergen Gül, Eda Açıkgöz, Mustafa Çakır, Nurettin Menges

**Affiliations:** † Science and Technology Research and Application Center (BITAM), Necmettin Erbakan University, 42100 Konya, Türkiye; ‡ School of Medicine, Van Yüzüncü Yil University, 65080 Van, Türkiye; § Faculty of Pharmacy, Van Yüzüncü Yil University, 65080 Van, Türkiye

## Abstract

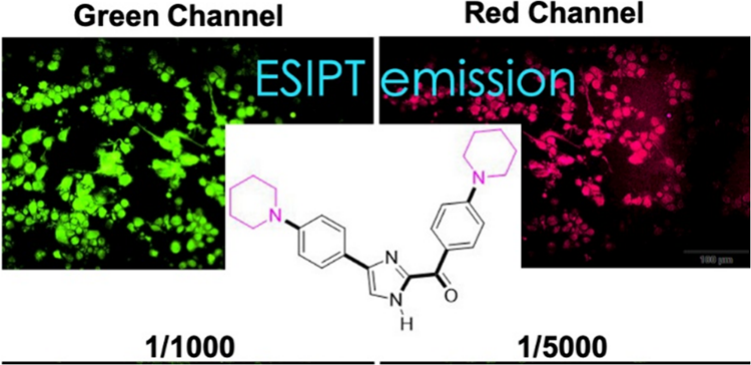

Excited-state intramolecular proton transfer (ESIPT)-based fluorescent molecules offer several exciting applications and are utilized most frequently as a cell imaging agent. Because of this, four distinct imidazole derivatives with ESIPT emission have been synthesized, and their fluorescence characteristics have been assessed in a variety of settings. Measurements using fluorescence spectroscopy have shown a promising candidate for cell staining, and potential candidate was specifically investigated for cell imaging uses in HT-29, MDA-MB-231, and HaCaT. Cytotoxicity of candidate molecule (**1d**) was analyzed using HT-29 and HaCaT cell lines, and at a dosage of 160 μM, HT-29 and HaCaT cell lines showed no signs of important cell toxicity. When spectroscopically measured, compound **1d** showed no fluorescence ability in phosphate-buffered saline (PBS) solution. However, after 8 h of incubation in several cell lines, excellent fluorescence characteristics were seen in the green and red filters.

## Introduction

Noninvasive fluorescent-based imaging agents mediated the visualization of changes at the cell and tissue level, and emerge as an important diagnostic method in medicine.^1^ Although tremendous advances have been made in fluorescence-based cell imaging techniques recently, efforts to develop fluorescent probes that target intracytoplasmic structures, which are less costly and more advantageous in terms of processing, enabling both *in vitro* and *in vivo* cell imaging, remain a major challenge in this area. Considering this point, fluorescent agents that enable quantitative *in vitro* and *in vivo* imaging provide a holistic approach to unbiased clinical assessments.^1,2a^ Cellular homeostasis, which changes based on dynamic fluctuations in organelles and biomolecules in the cell, is particularly important in the diagnosis and treatment of many disorders.^2b^

Fluorescent-based imaging agents have several applications and several distinct emission mechanisms, one of which is excited-state intramolecular proton transfer (ESIPT) emission.^3−6^ ESIPT emission covers a unique mechanism that results in two distinct emission bands in most cases.^7,8^ Large Stokes shift, possible biocompatibility, high cell penetration ability, and phototautomerization in excited state make it a more valuable candidate for special imaging processes including lysosome, DNA, and nucleus.^9−12^

**Figure 1:**
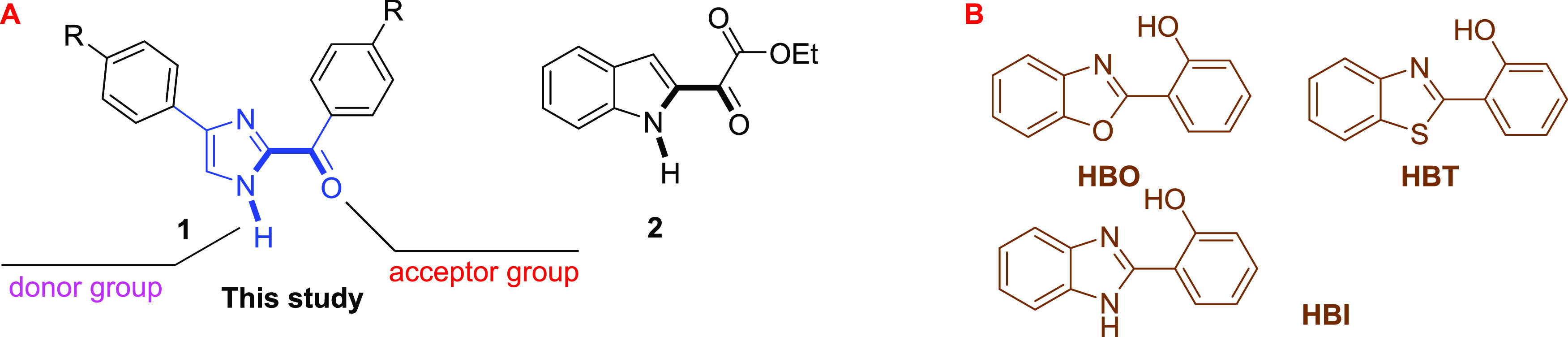
(A) Our defined heterocycles with ESIPT emission. (B) Well-known ESIPT-based heterocycles.

To enable ESIPT emission, the structure should include proton donor and acceptor functionalities that are close to each other *via* a 5- or 6-membered pseudo-ring system. In the literature, the most commonly structures are hydroxy-benzimidazole (HBI), hydroxy-benzoxazole (HBO), and hydroxy-benzothiazole (HBT) (Figure 1B). On the other hand, in the past few years, our research group found out that the carbonyl functional group at the C-2 position of imidazole **1** and indole derivatives **2**(13b) can make ESIPT emission (Figure 1A). These structures are logically arranged and include significant functional groups that might be relevant in future applications.^14,15^

In this study, we investigated imidazole compounds with ESIPT emission for cell imaging. As a result, numerous imidazole derivatives were synthesized, and their ESIPT emission potentials were measured. A search of those molecules produced a possible molecule for further cell imaging study. The staining pattern of the fluorescent agent created based on ESIPT in both fixed and living cells was examined using several cell lines such as HT-29 (colon cancer cell line), MDA-MB-231 (breast cancer cell line), and HaCaT (keratinocyte cell line) utilizing the candidate imidazole derivative. This is the first study to utilize our previously announced ESIPT-based fluorescent probe for cell imaging. In this study, we hope to understand the probe’s potential for cell imaging as well as to envision future applications such as varied analyte detection *in vitro* and *in vivo*.

## Experimental Section

### General Materials and Methods for Chemistry

^1^H- and ^13^C NMR spectra were recorded using a Varian NMR-400 MHz. ^1^H chemical shifts were referenced to internal standard TMS (δ 0.00 ppm) or deuterated solvents such as *d*_6_-DMSO and CDCl_3_. All chemical shifts (δ) are indicated in ppm, and *J* values are indicated in Hz. ^13^C NMR spectra were fully decoupled. The following patterns were designated as s, singlet; bs, broad singlet; d, doublet; dd, doublet of doublets; t, triplet; and m, multiplet. As a purification technique, column chromatography was performed on silica gel (60-mesh) and was followed with TLC that was made of Merck 0.2 mm silica gel 60 F_254_ analytical aluminum plates. Absorbance measurements were performed using Shimadzu UV-3600 Plus ultraviolet–visible–near-infrared (UV–vis–NIR) spectroscopy, and fluorescence measurements were performed utilizing Agilent Cary Eclipse spectrophotometer. Melting points were obtained on an X-4 digital melting point apparatus without correction. Solvents were evaporated at reduced pressure using a rotary vacuum evaporator.

### General Procedure for Synthesis of **1a**–**d**

One mmol portion of aryl methyl ketone was dissolved in 7 mL of 1,4-dioxane. The solution was then added with 2.5 mmol (0.275 g) of selenium dioxide (SeO_2_), and the reaction mixture was refluxed overnight in an oil bath. The TLC method was used to monitor the reaction’s completion. The mixture was separated from solid black Se^0^ using a filter and cooled at room temperature. On the other hand, 5 mmol (0.385 g) of ammonium acetate (CH_3_COONH_4_) was dissolved in 10 mL of ethanol and mixed at room temperature for 1 h. The solution was then mixed with 20 mL of ice water and stirred for 1 h. The solid was finally filtered and dried on phosphorus pentoxide.

#### Phenyl(4-phenyl-1*H*-imidazol-2-yl)methanone^1^ (**1a**)^13−15^

Light yellow solid, mp: 190 °C, yield: 68.5%. FTIR (ATR) cm^–1^:3647, 3269, 3092, 3061, 2980, 2888, 1669, 1615, 1597, 1570, 1020, 957, 935, 903, 868. ^1^H NMR (400 MHz, DMSO) δ 8.48 (d, *J* = 7.11 Hz, 2H, H-19 and H-15), 8.05 (s, 1H, H-8), 7.92 (d, *J* = 7.11 Hz, 2H, H-3 and H-5), 7.68 (t, *J* = 7.50 Hz, 1H, H-17), 7.60–7.58 (m, 2H, H-16 and H-18) 7.41 (t, *J* = 7.49 Hz, 2H, H-2 and H-6), 7.29 (t, *J* = 7.49 Hz, 1H, H-1). ^13^C NMR (100 MHz, DMSO) δ: 181.1, 144.7, 141.3, 136.3, 133.6, 132.5, 131.0, 129.2, 128.8, 128.1, 125.6, 120.9.
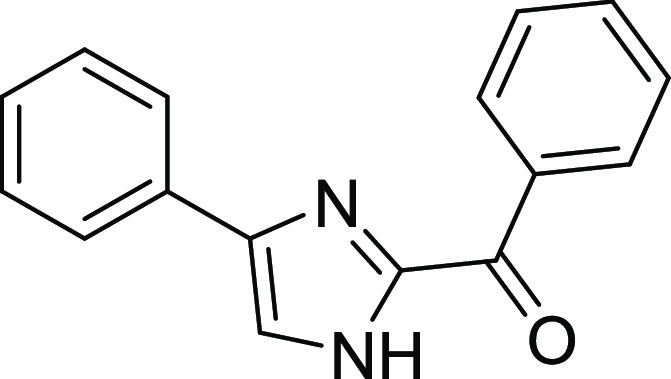


#### (4-Iodophenyl)(4-(4-iodophenyl)-1*H*-imidazol-2-yl)methanone (**1b**)

Light brown solid, mp: 218–221 °C. Yield 92%. ^1^H NMR (400 MHz, *d*_6_-DMSO) δ = 13.70 (bs, 1H, -NH), 8.31–8.28 (m, AA′BB′ system, 2H, Ar–H), 8.12 (d, J = 2.43 Hz, 1H, Ar–H), 7.99–7.96 (m, AA′BB′ system, 2H, Ar–H), 7.77–7.67 (m, 4H, Ar–H). ^13^C NMR (100 MHz, *d*_6_-DMSO) δ = 180.5, 145.0, 142.4, 137.8, 137.7, 135.6, 133.6, 132.7, 127.4, 119.8, 102.5, 93.3. HRMS (ESI) (M + H)^+^: C_16_H_31_I_2_N_2_O: 501.0859; found: 501.0843.
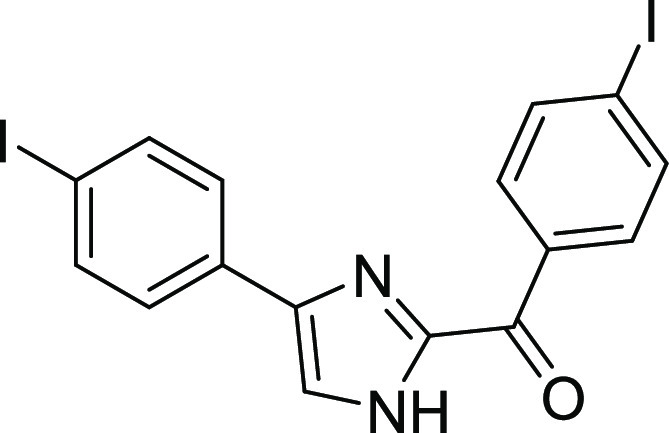


#### (4-Methoxyphenyl)(4-(4-methoxyphenyl)-1H-imidazol-2-yl)methanone (**1c**)^13−15^

Yellow solid, mp: 204–206 °C, yield: 87.5% FTIR (ATR) cm^–1^: 3264, 3095, 2980, 2840, 1608, 1595, 1564, 1511, 1026, 972, 957, 903, 864. ^1^H NMR (400 MHz, DMSO) δ: 8.50–8.48 (m, AA′BB′ system, 2H, Ar–H), 7.94 (s, 1H, H-8), 7.87–7.86 (m, AA′BB′ system, 2H, Ar–H), 7.13–7.11 (m, AA′BB′ system, 2H, Ar–H), 7.00–6.68 (m, AA′BB′ system, 2H, H-2, H-6), 3.87 (s, 3H, OMe), 3.78 (s, 3H, OMe). ^13^C NMR (100 MHz, DMSO) δ: 179.1, 163.9, 159.5, 144.1, 133.5, 128.8, 127.2, 124.2, 124.1, 119.8, 114.7, 114.3, 56.1, 55.6.
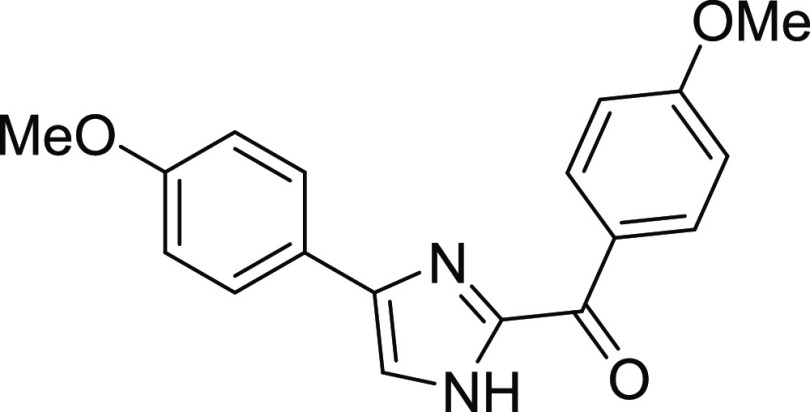


#### (4-(Piperidin-1-yl)phenyl)(4-(4-(piperidin-1-yl)phenyl)-1*H*-imidazol-2-yl)methanone (**1d**)

Yellow solid, mp: 219–220 °C, yield: 69%. ^1^H NMR (400 MHz,*d*_6_-DMSO) δ = 13.23–13.16 (bs, 1H, -NH), 8.60–54, 8.46–8.42 (m, AA′BB′ system, 2H, Ar–H), 7.76–7.74, 7.52–7.50 (m, 3H, Ar–H), 7.04–6.97 (m, AA′BB′ system, 2H, Ar–H), 6.95–6.92 (m, AA′BB′ system, 2H, Ar–H), 3.43–3.41 (m, 2H), 3.22–3.17 (m, 1H), 3.15–3.13 (m, 2H), 1.61–1.56 (m, 6H). ^13^C NMR (100 MHz, *d*_6_-DMSO) δ = 178.5, 154.2, 151.1, 145.6, 143.2, 133.4, 126.9, 124.8, 116.1, 115.7, 113.1, 49.9, 48.2, 48.1, 25.7, 25.5, 24.5, 24.4, HRMS (ESI) (M + H)^+^: C_26_H_31_N_4_O:415.2498, found: 415.2481.
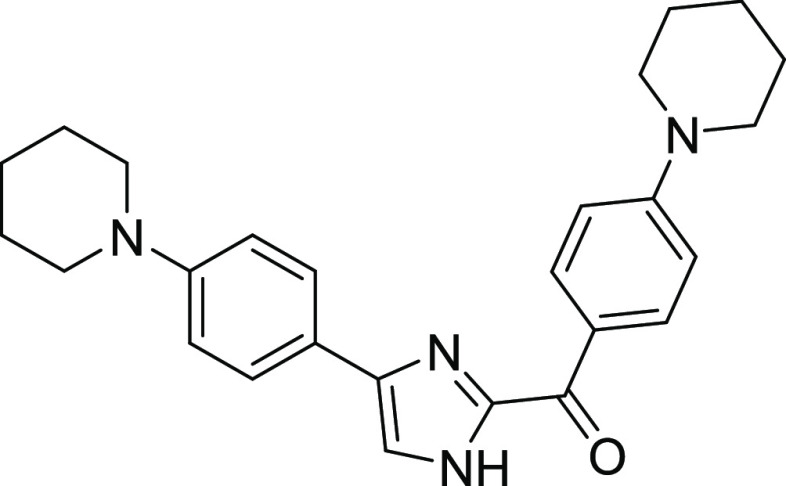


### General Materials and Methods for Cell Imaging

MDA-MB-231 (human breast adenocarcinoma), HT-29 (human colon adenocarcinoma), and HaCaT (human keratinocyte) cell lines were purchased from ATCC (Manassas) and cultured in Dulbecco’s Modified Eagle’s Medium (DMEM) (Gibco, Germany) supplemented with 10% fetal bovine serum (FBS) (Gibco), 100 U/mL penicillin, and 100 μg/mL streptomycin (Gibco). All of these cell lines were maintained with an incubator under standard cell conditions (37 °C, 5% CO_2_, and 95% air atmosphere).^16^ The general morphology of the cells, contrasting the cell nuclei from the cytoplasm, and the fluorescence of the molecule in the cells were observed by inverted microscope, light microscope, and fluorescence microscope, respectively.

To prepare for imaging, the cells were seeded on a coverslip coated with poly-l-Lysine according to the protocol used in the previous study.^16^ Briefly, the cells were cultured for 24 h to obtain their normal morphological shape. Then, the cells were fixed with 4% paraformaldehyde (PFA) for 15 min, washed with phosphate-buffered saline (PBS) (3 × 5 min), and viewed with an inverted microscope. In the hematoxylin–eosin (H&E) staining protocol performed to provide nucleus–cytoplasm contrast, cells were immersed in hematoxylin (1 min) and eosin (30 s), respectively, and covered with cell mounting medium and visualized with a light microscope.

The intracellular distribution of molecule **1d** was investigated using a fluorescence microscope. To determine both the fluorescence potential and the optimum concentration of molecule **1d**, MDA-MB-231 cells were incubated with different concentrations of molecule **1d** (20, 10, 2 μM) for 30 min, and then the cells were washed with PBS and visualized using blue, green, and red channels. To reveal the compartments stained by molecule 1d in different fixed cells (HT-29, MDA-MB-231, and HaCaT), typically cells were fixed with 4% PFA for 15 min and washed with PBS, and cells were stained with molecule 1d for 30 min and observed under a fluorescence microscope. To measure the live cell imaging (without fixation) potential of molecule d and to detect the areas it has stained inside the cell, HT-29 and HaCaT cells were incubated with molecule d for 8 h, washed with PBS, and visualized in both green and red channels with a fluorescence microscope.

### Cytotoxicity Test for Molecule **1d**

HaCaT keratinocyte cells were cultured in DMEM medium with 10% FBS and 1% penicillin/streptomycin, and HT-29 colon cancer cells were grown in RPMI 1640 medium with 10% FBS and 1% penicillin/streptomycin. ESIPT was prepared as 1 molar amount in DMSO. Cells were grown in 96-well plates at a density of 5 × 10^3^. It was applied to cells at various conditions for 24 h. The cell toxicity using MTT (3-(4,5-dimethylthiazol-2-yl)-2,5-diphenyltetrazolium bromide) was then quantified and assessed with a plate reader.^18^

### Statistical Analysis

Paired Student’s *t* test was used to compare two groups to determine cell imaging data measurements. One-way ANOVA and Dunnett’s multiple comparison tests were used to compare more than two groups. *P* values less than 0.05 were considered to be statistically significant.

## Results and Discussion

### Design and Synthesis

Figure 1A,B demonstrates that compounds 1 and 2 have several benefits over the popular skeletons in Figure 1B. Compounds 1 and 2 include a carbonyl group, and compound 2 has additional ester functionality. Furthermore, substituents on the benzene ring and potential replacements on the free nitrogen atom in compounds 1 and 2 may give substantial advantages when researchers strive to develop alternative functionality for ESIPT-based emitting compounds. These benefits are confined to compounds 1 and 2 and do not apply to the compounds mentioned in Figure 1B. Customizing the functional groups of compounds 1 and 2 may not influence the ESIPT mechanism, as seen in our previous study.^15^

**Scheme 1:**
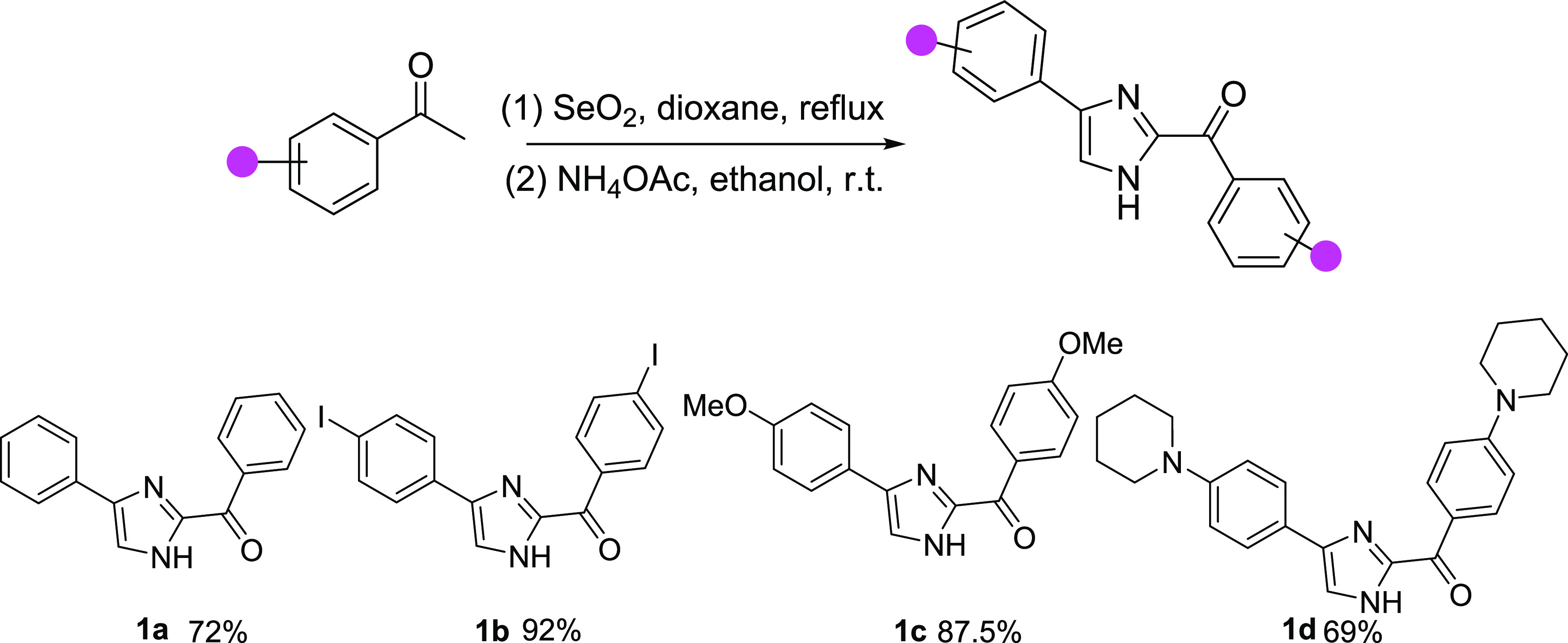
Synthesis of Imidazole Derivatives That Have ESIPT Emission

For the imidazole ring, we have used our previous synthetic method.^13^ In this method, acetyl functionality on the benzene ring was oxidized using SeO_2_ following cyclization with NH_4_OAc in ethanol *via* two-step and one-pot reaction protocol that makes this protocol highly applicable (Scheme 1).

Four different molecules were synthesized to identify the best candidates for the study. Derivatives containing unsubstituted benzene **1a**, para-iodo benzene **1b**, para methoxybenzene **1c**, and para piperidine benzene **1d** groups were obtained with good yields.

We have stated in our previous studies that electron-donating groups in the benzene ring of molecules are important for fluorescence. In this study, fluorescence effects were investigated using both OMe **1c** and piperidine group **1d**. In addition, a para-iodo benzene derivative **1b** was synthesized to examine the heavy atom effect.

### UV–Vis and Fluorescence Spectra

According to the spectroscopic investigation, the imidazole derivative with an unsubstituted benzene ring (**1a**) displays an emission band of 459 nm while having no absorption in the visible area. The presence of an iodine atom in the para position of the benzene ring 1b produced an emission band at 480 nm with a red shift of around 21 nm (**1b**) (Table 1). The emission band of an imidazole derivative with an electron-donating group such as OMe at the para position (**1c)** was measured to be 494 nm with a red shift of up to 40 nm. Finally, compared with other derivatives, the derivative containing a piperidine ring at the para position of benzene (**1d**) exhibited the most significant red shift, with an emission band at 590 nm (in DMSO solvent).

**Table 1:** Absorbance and Fluorescence Max Values for Compounds **1a**–**d**^a^

molecule	absorbance (λ_max_, nm)	fluorescence (λ_em_, nm)
**1a**	348	459
**1b**	354	480
**1c**	366	494
**1d**	385	460, 590

a 30 μM in DMSO.

Our group was the first to publish on the ESIPT emission theory of this skeleton. As a result of these investigations, our group discovered the optimal emission value for the corresponding skeleton. As a result, we believe that it might be an ideal candidate for cell imaging research.

**Figure 2:**
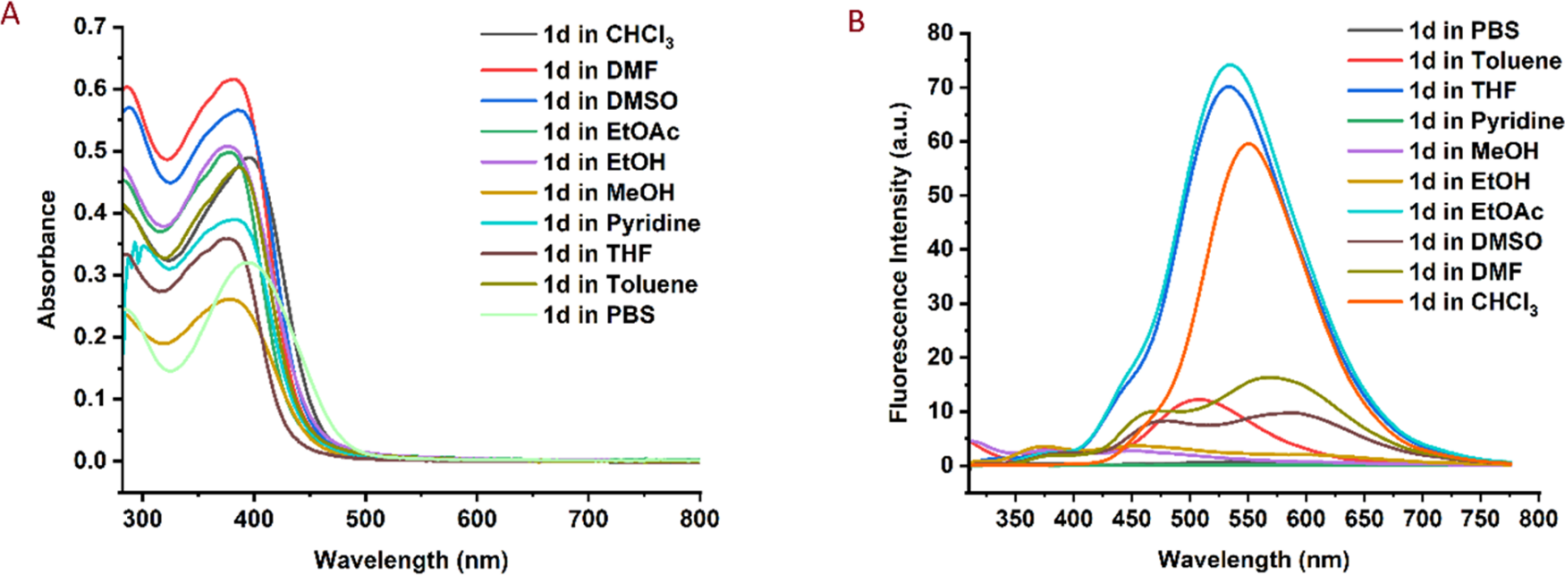
(A) Absorbance and (B) fluorescence spectra for **1d.** Concentration: 30 μM, excitation: 280 nm, PMT: 500 V, and slit width: 5 nm. PBS: phosphate-buffered saline solution.

**Figure 3:**
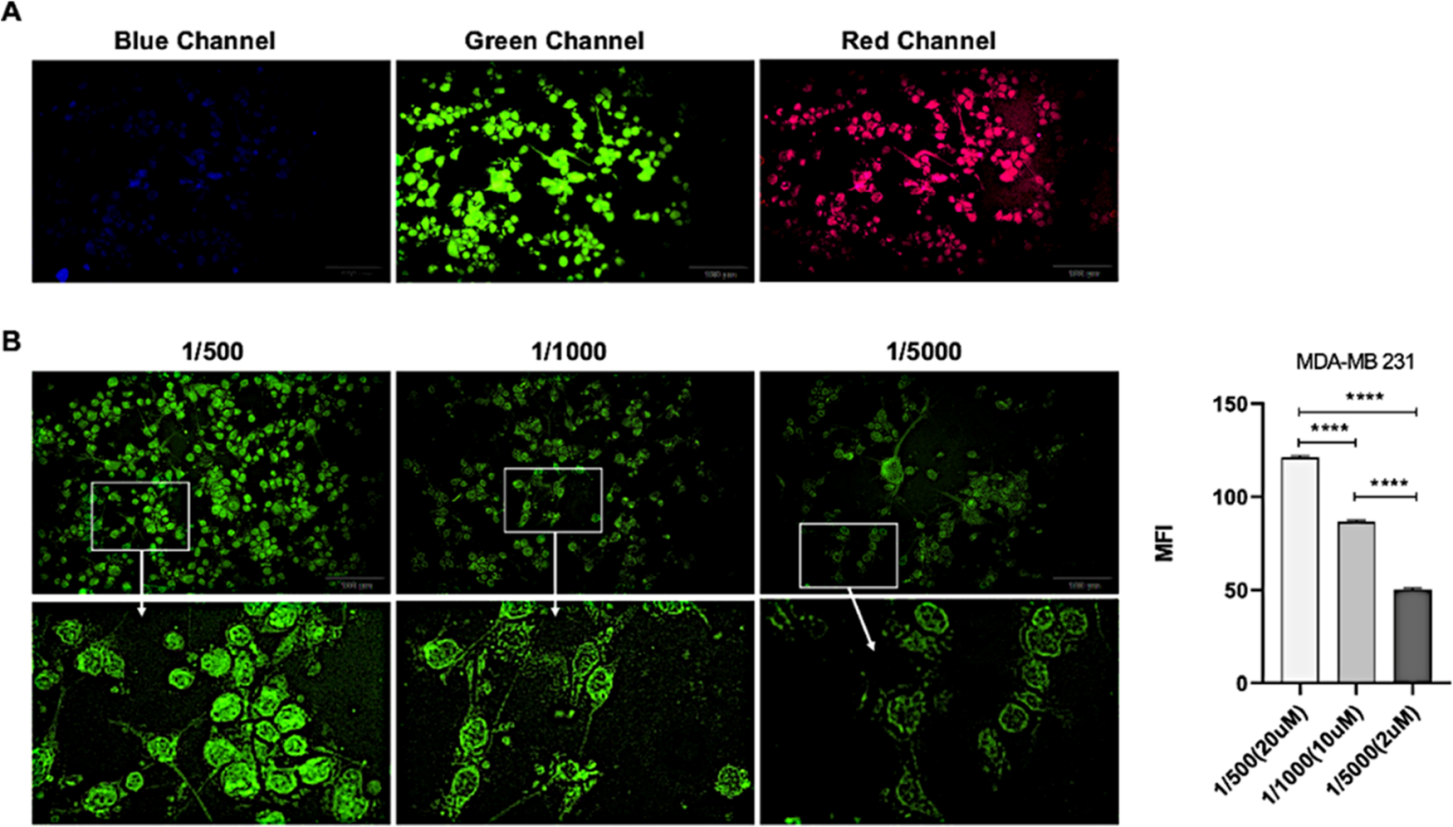
Immunofluorescence imaging of cells incubated with Molecule d. (A) MDA-MB-231 cells were incubated with 20 μM concentration of molecule **1d** and visualized in 3 different channels (blue, green, and red). (B) MDA-MB-231 cells were stained with different concentrations of molecule **1d** (20, 10, and 2 μM) and photographed with green channels (scale bar: 100 and 50 μm).

The selected chemical’s behavior in various solvents was also studied. Different solvents were used for this purpose (Figure 2A–B, and Table 2), and the absorbance value did not vary significantly even when employing cell media PBS. Furthermore, none of the absorbances in the different solutions revealed the value of the visible area, demonstrating that the molecule does not interact differently with different solvents in the ground state. However, the solvents used have an influence on the fluorescence spectrum of **1d**. When compared with the other solvents, MeOH and EtOH, like many ESIPT-based luminescent compounds, did not display significant fluorescence properties. The fluorescence intensity was highest in EtOAc, THF, and CHCl_3_ solutions, which had 0.710, 0.767, and 0.557 quantum yields, respectively, with maximum values of 535, 533, and 553 nm. In DMSO and DMF solvents, two distinct bands with modest fluorescence intensity were identified, which might be because of solvent interactions due to the nature of solvent and nonbonding electrons. The fact that these two solvents give two bands, unlike the others, is due to high-energy excitation because it was observed that the fluorescence emission decreased to a single band as the excitation energy decreased up to 440 nm (see Figures S1 and S2). The fluorescence intensity of the solution generated in toluene, an aromatic molecule, was equally modest, with a max value of 507 nm with 0.132 quantum yield. It is worth noting that **1d** in PBS solution did not show any fluorescence intensity, as predicted because of the nature of ESIPT emission. However, when the molecule of **1d** was introduced into the cell, we found considerable green and red fluorescence images on the microscopy, even at 2 μM solution (Figure 3), which means that **1d** can penetrate the cell and have some interactions with cell organelles.

**Table 2:** Solvent Screening on Absorbance and Fluorescence Spectra for **1d**^a^

solvent	absorbance (λ_max_, nm)	fluorescence (λ_em_, nm)	quantum yield^b^
EtOH	379	450	0.048
MeOH	381	449	0.052
pyridine	388	-c	0.003
THF	378	533	0.767
toluene	386	507	0.132
DMF	382	468, 570	0.178
EtOAc	380	535	0.710
CHCl_3_	398	553	0.557
DMSO	385	460, 590	0.106
PBS	398	-c	0.015

a Concentration: 30 μM, excitation: 280 nm, PMT: 500 V, slit width: 5 nm.

b Fluorescein was used as a standard; PBS: phosphate-buffered saline.

c Not applicable.

### Cell Imaging Studies

To evaluate the potential biological application of molecule **1d** in cell imaging, fluorescent microscopy experiments were performed using fixed MDA-MB-231 cells. In the first step, (i) in which channels molecule d generates a detectable fluorescent signal and (ii) optimum concentrations of **1d** were investigated. Three different channels (blue, green, and red) and concentrations (20, 10, and 2 μM) were tested. MDA-MB-231 cells were incubated with molecule d for 30 min, and cells were visualized using the blue, green, and red channels. As seen in Figure 3A, a weak fluorescent signal was obtained in the blue channel, but a strong fluorescent signal was detected in the green and red channels (*p* < 0.0001).

When the staining pattern of the cells is evaluated specifically, it cannot be distinguished exactly which compartments are stained in the cell due to the intense fluorescent signal. To gain a deeper insight into the structures with which molecule 1d specifically interacts in cells, MDA-MB-231 cells were stained using different concentrations of molecule **1d** (20, 10, and 2 μM). Measurements with ImageJ showed that the mean fluorescent intensity (MFI) values were statistically significant due to the increasing concentration of molecule d (*p* < 0.0001).

Considering its distribution in the cell and the appropriate fluorescent signal, it has been determined that the 10 μM concentration of **1d** was optimum (Figure 3B), and this concentration was used in both fixed and living cells.

**Figure 4:**
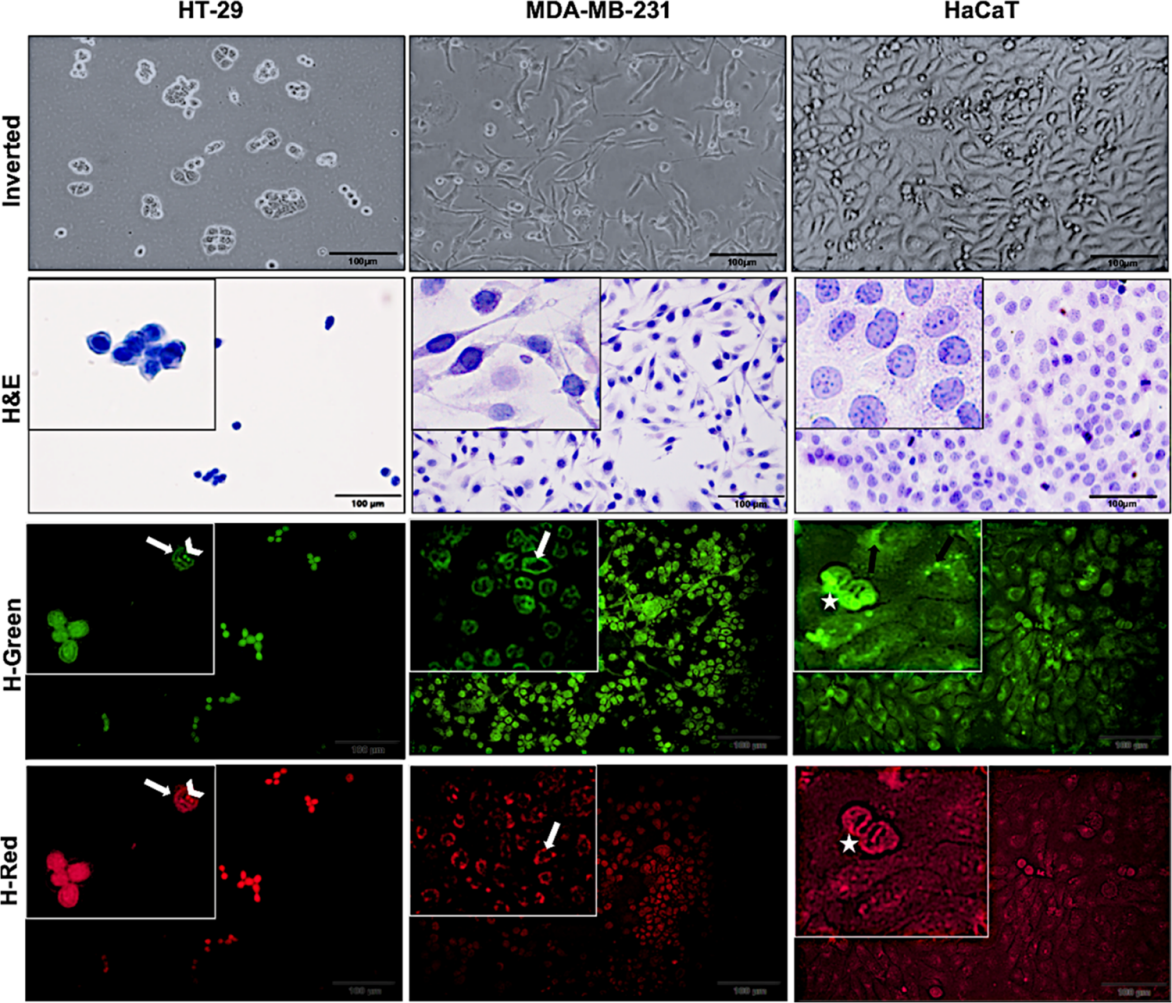
Image of HT-29, MDA-MB-231, and HaCaT cells with different microscopes (inverted, light, and fluorescence microscope). White arrow, black arrow, and white asterisk indicate cell membrane, subnuclear region, and dividing cells, respectively (scale bar: 100 μm).

To explore the fluorescent staining pattern of **1d** in the fixed cells, HT-29, MDA-MB-231, and HaCaT cells were incubated for 30 min and viewed under an Olympus BX53 light microscope with an attached DP74 camera. Furthermore, the cells were examined under both an inverted microscope and a light microscope to confirm the stained areas inside the cell (Figure 4). In particular, H&E staining clearly reveals the shape of the cell mediated the differentiation of cytoplasm and nucleus in the cell.

As seen in Figure 4, green and red fluorescent signals were obtained in HT-29, MDA-MB-231, and HaCaT when cells were stained with molecule **1d**. Considering the localizations in the cell, it was determined that molecule d fluorescence was intense in the cell membrane (white arrow) and nucleolus (white arrowhead) structure of HT-29 cells. In MDA-MB-231 cells, a strong fluorescence was obtained in the nuclear membrane. In the image presented in Figure 4, a different staining pattern was displayed in HaCaT cells compared with the others. It was determined that molecule **1d** intensely fluoresced in the subnuclear region (black arrow). Interestingly, in HaCaT cells, it was noted that dividing cells (white asterisks) were stained extensively with **1d**. These results revealed that **1d** specifically stains cell membranes, nuclei, and subnuclear areas in cells.

Fluorescent-based live cell imaging allows real-time observation of the interaction processes between cellular structures and molecules, providing more insight into cellular processes than a snapshot provided by imaging fixed cells.

**Figure 5:**
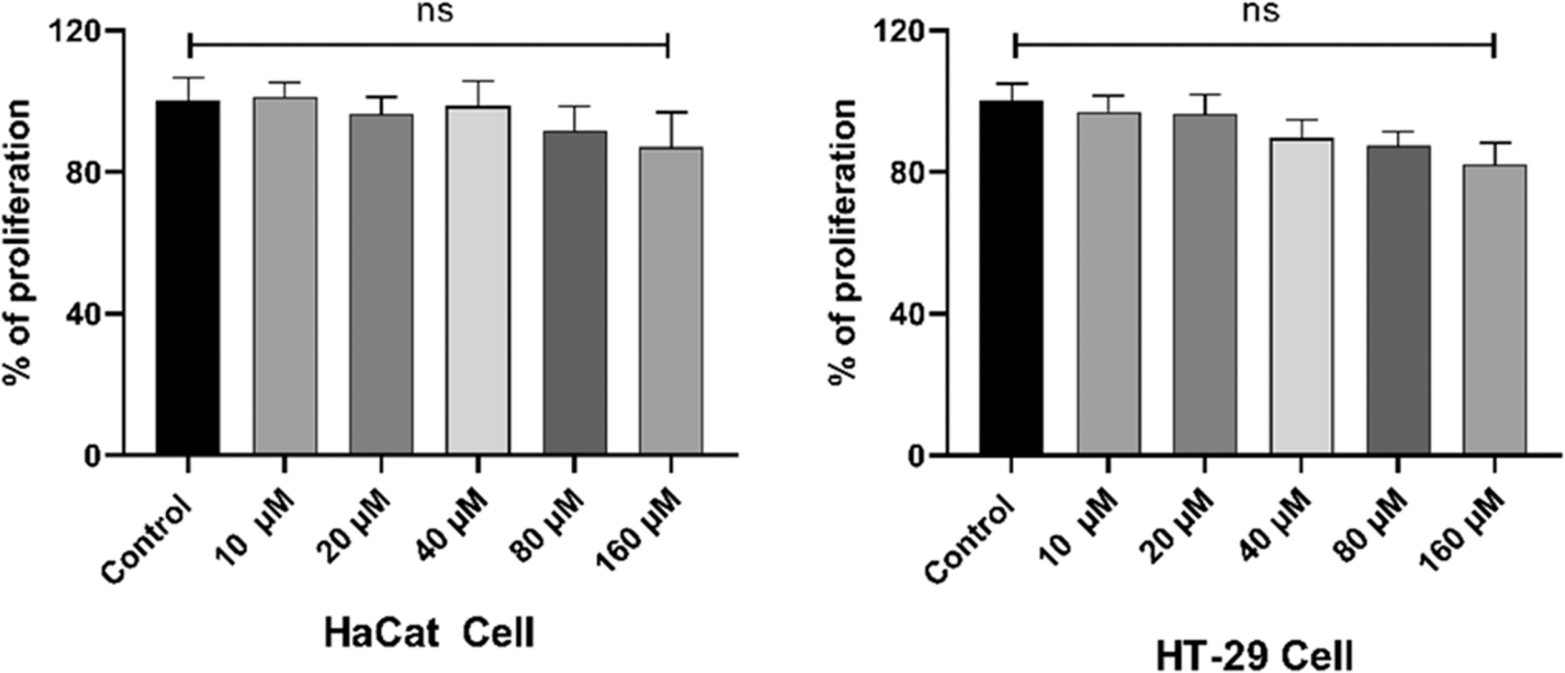
Cytotoxicity effect of compound **1d** on two different cell lines.

A chemical cannot be used for live imaging if it produces any harmful side effects. For this purpose, we first tested whether compound **1d** we used in our study had a toxic effect on the cell. The dose range of 10–160 μm has no cytotoxic effect on HT-29 and HaCaT cells during 24 h (Figure 5).

**Figure 6:**
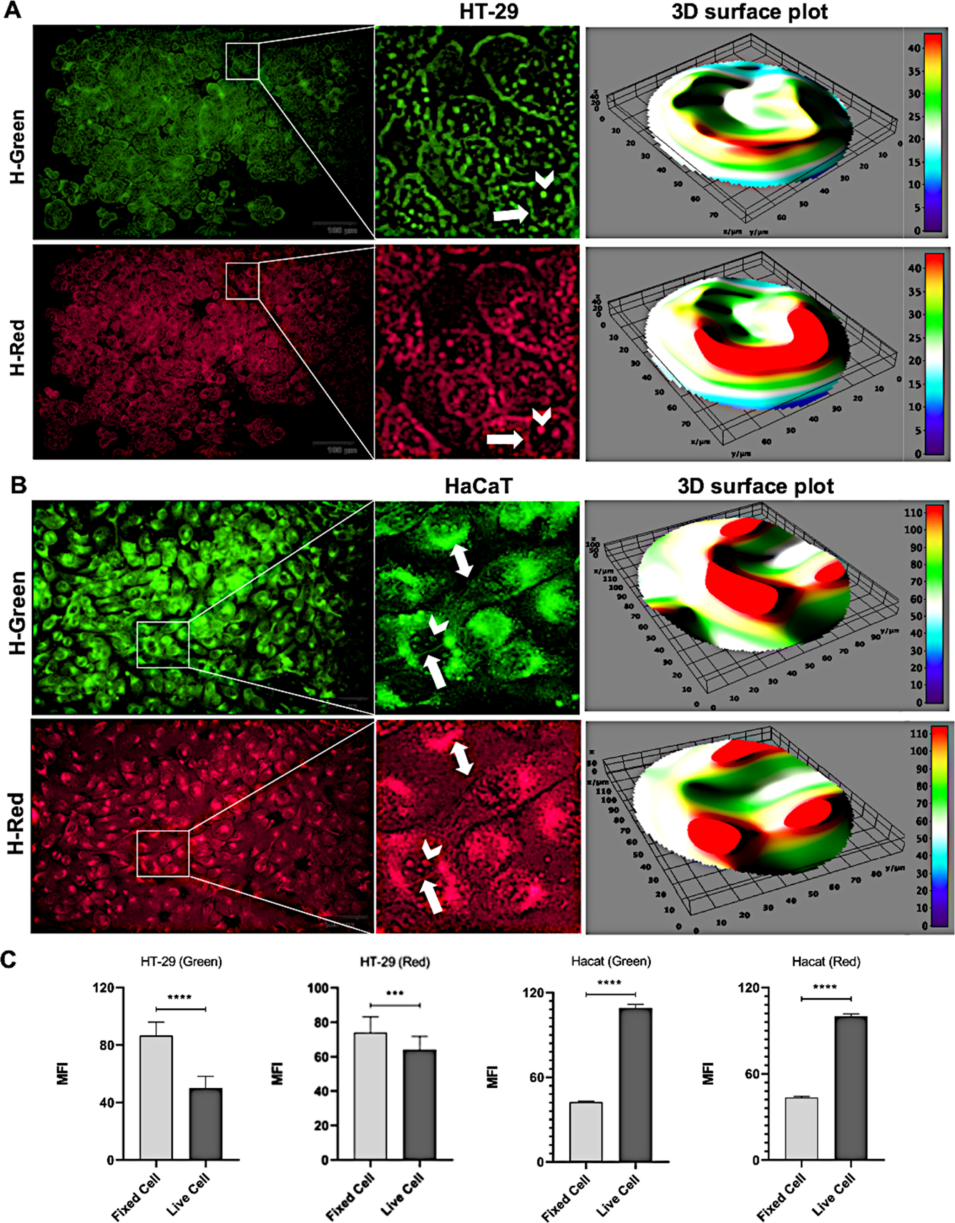
Live imaging of molecule d in cells. HT-29 (A) and HaCaT (B) cells were incubated with molecule d for 8 h and visualized in the green channel and red channel, and a 3D surface plot was obtained. (C) Both fixed and live imaging graphs of HT-29 and HaCaT cells. The white arrow, white arrowhead, and double-headed white arrow point to the cell membrane, nucleolus, and subnucleus, respectively (scale bar: 100 and 50 μm).

In light of these data, encouraged by the fluorescent images obtained from fixed cells, we evaluated the potential for the use of **1d** in live cell imaging. HT-29 and HaCaT cells were incubated with **1d** for 8 h, and then the cells were washed with PBS and viewed under a fluorescent microscope. Similar staining patterns were obtained in live imaging, confirming the results in fixed cells (Figure 6). In HT-29 cells, the fluorescence intensity was quite prominent in the cell membranes and nuclei (Figure 6A). Mean fluorescence intensity (MFI) values of molecule **1d** were measured for both fixed and live cells using ImageJ. MFI values were higher in fixed cells compared with live cells in HT-29 green channel (*p* < 0.0001) and red channel (*p* < 0.001) (Figure 6C). However, the staining pattern in living cells was observed to be more specific. The MFI value was higher in the fixed cells, probably due to some nonspecific weak staining in the cytoplasm. Three-dimensional (3D) surface plot analyses of HT-29 cells showed that the intensity of fluorescent staining was in the nuclear membrane and nucleolus regions (Figure 6A).

Live imaging of HaCaT cells is both more intense and highly specific with fluorescence intensity in the green and red channels compared with fixed cells (Figure 6). Furthermore, MFI values were obtained higher in live cells than in fixed cells (*p* < 0.0001) (Figure 6C). In addition to a prominent nuclear membrane and nucleolus fluorescence staining, the intensity of the staining in the subnucleus structure is quite remarkable. Condensed fluorescence areas were also observed in certain regions in the HaCaT cytoplasm (double-headed white arrow). This staining pattern in the cytoplasm indicates that a specific organelle or molecule is stained in the cell. As seen in Figure 6B, surface plot analyses of HaCaT cells showed that the fluorescence intensity was particularly prominent in the subnucleus region.

To summarize, the absorbance and fluorescence properties of four distinct imidazole derivatives, the ESIPT properties of which were recently discovered by our research group, were studied. The imaging capabilities of **1d**, which was shown to have the greatest shift to the red area, were studied in both healthy (HacaT) and malignant cell lines (HT-29 and MDA-MB-231). Furthermore, **1d**’s cytotoxicity was tested, and the cell viability of HT-29 and HaCaT at 160 μM concentration was determined to be at least 80%. Compound **1d** emitted at a high level and fluoresced in both green and red filters. Furthermore, in terms of organelle staining, it was discovered that it stained distinct locations in healthy and malignant cells, which is the most relevant concern. The chosen ESIPT-based skeleton was employed for the first time in the literature in cell-staining investigations, and it was discovered that it had significant potential. In future experiments, we will study the mechanistic explanations of the organelles dyed by compound **1d** and report on more specific findings.

## Supporting Information Available

Fluorescence; NMR; and HPLC spectra for compounds **1-d** (PDF)

## References

[ref1] Sedgwick A. C.; Wu L.; Han H. H.; Bull S. D.; He X. P.; James T. D.; Yoon J.; et al. Excited-State Intramolecular Proton-Transfer (ESIPT) Based Fluorescence Sensors and Imaging Agents. Chem. Soc. Rev. 2018, 47 (23), 8842–8880. 10.1039/C8CS00185E. 30361725

[ref2] aIsherwood B.; Timpson P.; McGhee E. J.; Anderson K. I.; Canel M.; Serrels A.; Brunton V. G.; Carragher N. O. Live Cell in Vitro and in Vivo Imaging Applications: Accelerating Drug Discovery. Int. J. Pharm. 2011, 3 (2), 141–170. 10.3390/pharmaceutics3020141. PMC3864231 24310493

[ref3] Wang L.; Frei M. S.; Salim A.; Johnsson K. Small-Molecule Fluorescent Probes for Live-Cell Super-Resolution Microscopy. J. Am. Chem. Soc. 2019, 141 (7), 2770–2781. 10.1021/jacs.8b11134. 30550714

[ref4] Gong F.; Zeng D.; Zhu H.; Qian Y.; He L.; Xia J.; Cao Z. A Solvent-Assisted ESIPT Fluorescent Dye for F–/Ag+ Sensing and High-Resolution Imaging of the Cilia in Live Cells. Anal. Bioanal. Chem. 2021, 413 (25), 6343–6353. 10.1007/s00216-021-03590-3. 34378069

[ref5] Chen Y.; Fang Y.; Gu H.; Qiang J.; Li H.; Fan J.; Chen X.; et al. Color-unable and ESIPT-Inspired Solid Fluorophores Based on Benzothiazole Derivatives: Aggregation-Induced Emission, Strong Solvatochromic Effect, and White Light Emission. ACS Appl. Mater. Interfaces 2020, 12 (49), 55094–55106. 10.1021/acsami.0c16585. 33215923

[ref6] Chen M.; Liang Z.; Zeng G.; Wang Y.; Mai Z.; Chen X.; Wu G.; Chen T. An ESIPT-Based NIR-Emitting Ratiometric Fluorescent Probe for Monitoring Hydrogen Peroxide in Living Cells and Zebrafish. Dyes Pigm. 2022, 198, 109995 10.1016/j.dyepig.2021.109995.

[ref7] Azarias C.; Budzák Š.; Laurent A. D.; Ulrich G.; Jacquemin D. Tuning ESIPT Fluorophores into Dual Emitters. Chem. Sci. 2016, 7 (6), 3763–3774. 10.1039/C5SC04826E. 29997864 PMC6008603

[ref8] Mutai T.; Sawatani H.; Shida T.; Shono H.; Araki K. Tuning of Excited-State Intramolecular Proton Transfer (ESIPT) Fluorescence of Imidazo [1, 2-a] pyridine in Rigid Matrices by Substitution Effect. J. Org. Chem. 2013, 78 (6), 2482–2489. 10.1021/jo302711t. 23405828

[ref9] Dahal D.; McDonald L.; Bi X.; Abeywickrama C.; Gombedza F.; Konopka M.; Paruchuri S.; Pang Y. An NIR-Emitting Lysosome-Targeting Probe with Large Stokes Shift via Coupling Cyanine and Excited-State Intramolecular Proton Transfer. Chem. Commun. 2017, 53 (26), 3697–3700. 10.1039/C7CC00700K. PMC5492890 28294245

[ref10] McDonald L.; Dahal D.; Konopka M.; Liu Q.; Pang Y. An NIR Emitting Styryl Dye with Large Stokes Shift to Enable Co-Staining Study on Zebrafish Neuromast Hair Cells. Bioorg. Chem. 2019, 89, 103040 10.1016/j.bioorg.2019.103040. 31195328 PMC6656593

[ref11] Abeywickrama C. S.; Bertman K. A.; Mcdonald L. J.; Alexander N.; Dahal D.; Baumann H. J.; Pang Y.; et al. Synthesis of Highly Selective Lysosomal Markers by Coupling 2-(2′-hydroxyphenyl) Benzothiazole (HBT) with Benzothiazolium Cyanine (Cy): The Impact of Substituents on Selectivity and Optical Properties. J. Mater. Chem. B 2019, 7 (47), 7502–7514. 10.1039/C9TB01672D. 31712794

[ref12] Tang L.; Tian M.; Chen H.; Yan X.; Zhong K.; Bian Y. An ESIPT-Based Mitochondria-Targeted Ratiometric and NIR-Emitting Fluorescent Probe for Hydrogen Peroxide and Its Bioimaging in Living Cells. Dyes Pigm. 2018, 158, 482–489. 10.1016/j.dyepig.2017.12.028.

[ref13] aKuzu B.; Tan M.; Ekmekci Z.; Menges N. A Novel Structure for ESIPT Emission: Experimental and Theoretical Investigations. J. Photochem. Photobiol., A 2019, 381, 111874 10.1016/j.jphotochem.2019.111874.

[ref14] Kuzu B.; Ekmekci Z.; Tan M.; Menges N. Excited State Intramolecular Proton Transfer (ESIPT)-Based Sensor for Ion Detection. J. Fluoresc. 2021, 31 (3), 861–872. 10.1007/s10895-021-02716-1. 33772405

[ref15] Kuzu B.; Tan M.; Ekmekci Z.; Menges N. A Novel Fluorescent Sensor Based on İmidazole Derivative for Fe3+ Ions. J. Lumin. 2017, 192, 1096–1103. 10.1016/j.jlumin.2017.08.057.

[ref16] Güler G.; Acikgoz E.; Yavasoglu N. Ü. K.; Bakan B.; Goormaghtigh E.; Aktug H. Deciphering the biochemical similarities and differences among mouse embryonic stem cells, somatic and cancer cells using ATR-FTIR spectroscopy. Analyst 2018, 143 (7), 1624–1634. 10.1039/C8AN00017D. 29497718

[ref18] Acikgoz E.; Cakir M.; Guven M.; Oktemet G. Metformin Eliminates CD133/CD44 Prostate Cancer Stem Cells Via Cell Cycle Arrest and Apoptosis. Eurasian J. Med. Oncol. 2021, 5 (4), 298–304. 10.14744/ejmo.2021.11927.

