# Supplementary material for: Design and Synthesis of ESIPT-Based Imidazole Derivatives for Cell Imaging
Source: ACS Omega. 2024 May 27;9(23):24291–8. doi: 10.1021/acsomega.3c09822 (PMC11171098; doi:10.1021/acsomega.3c09822)
Supplement: Supplementary file 1 — ao3c09822_si_001.pdf [file ao3c09822_si_001.pdf]

SUPPORTING INFORMATION

Design and Synthesis of ESIPT-Based Imidazole Derivatives for Cell Imaging

Sergen Gül,[1] Eda Açıkgöz,[2] Mustafa Çakır,[2] Nurettin Menges*,[1,3]

[1]Science and Technology Research and Application Center (BITAM), Necmettin Erbakan University, 42100, Konya, Türkiye

[2]School of Medicine, Van Yüzüncü Yil University, 65080, Van, Türkiye

[3] Faculty of Pharmacy, Van Yüzüncü Yil University, 65080, Van, Türkiye

***Corresponding author**: nurettin.menges@erbakan.edu.tr

Contents

Excitation screening figures of **1d** in different excitation values S2

NMR spectra for synthesized compounds (**1a-d**) S3

HPLC chromatogram for **1d** S8

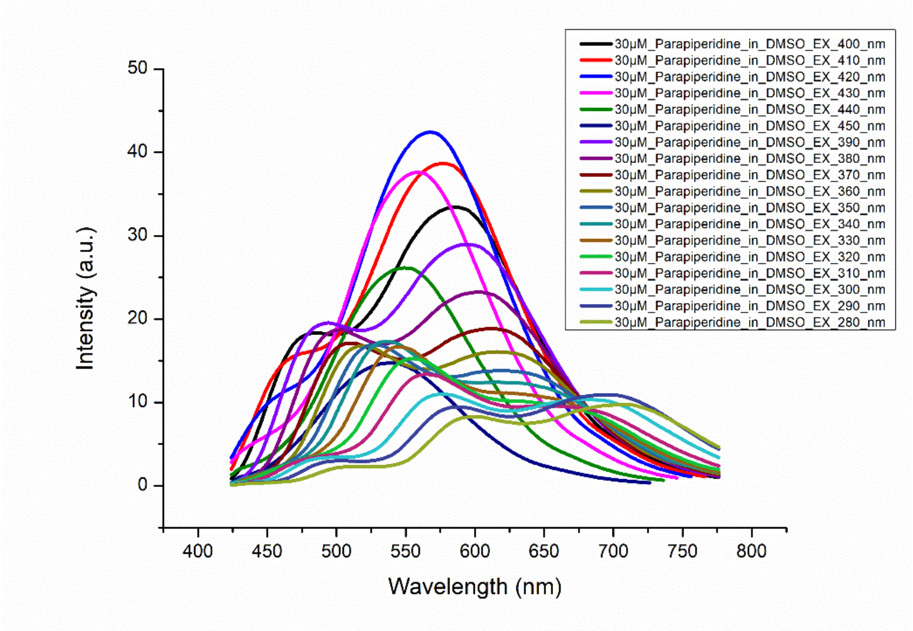

Figure S1. Fluorescence measurement of **1d** in DMSO using different excitation values. Concentration: 30 μM, PMT:500 V, Slit width: 5 nm

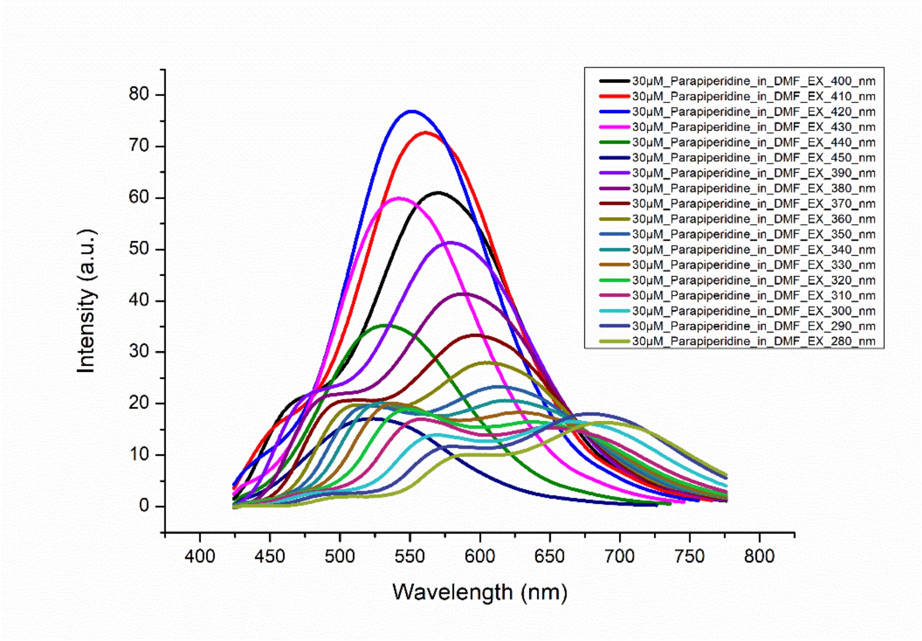

Figure S2. Fluorescence measurement of **1d** in DMF using different excitation values. Concentration: 30 μM, PMT:500 V, Slit width: 5 nm

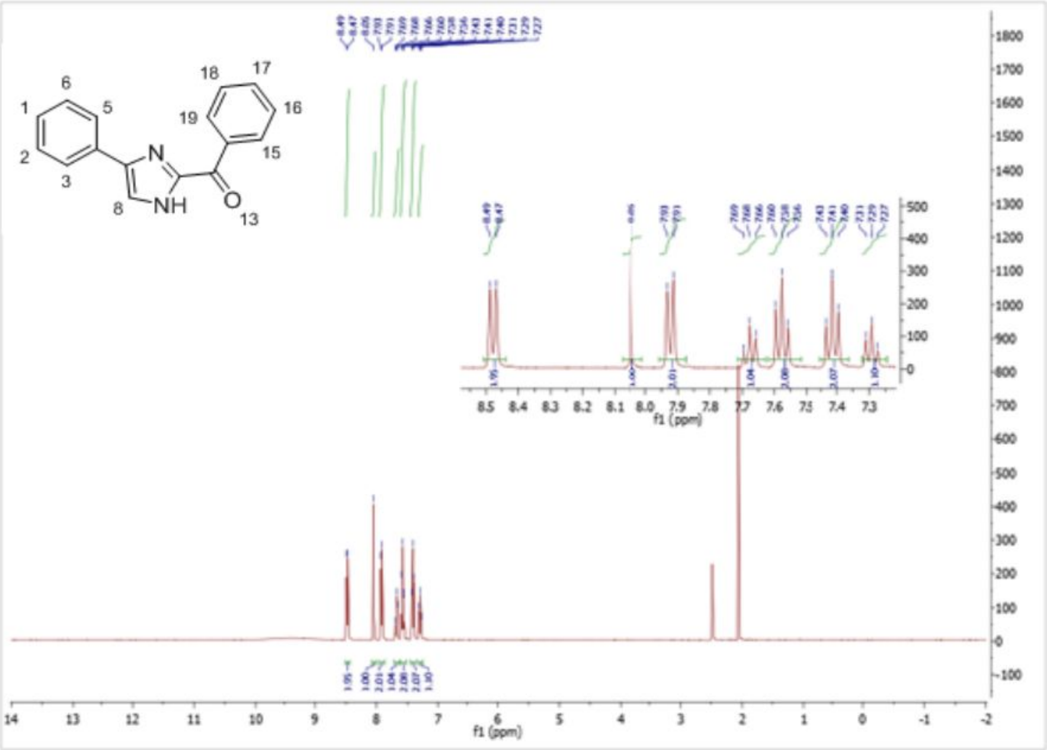

Figure S3. ^{1}H-NMR spectrum for compound 1a

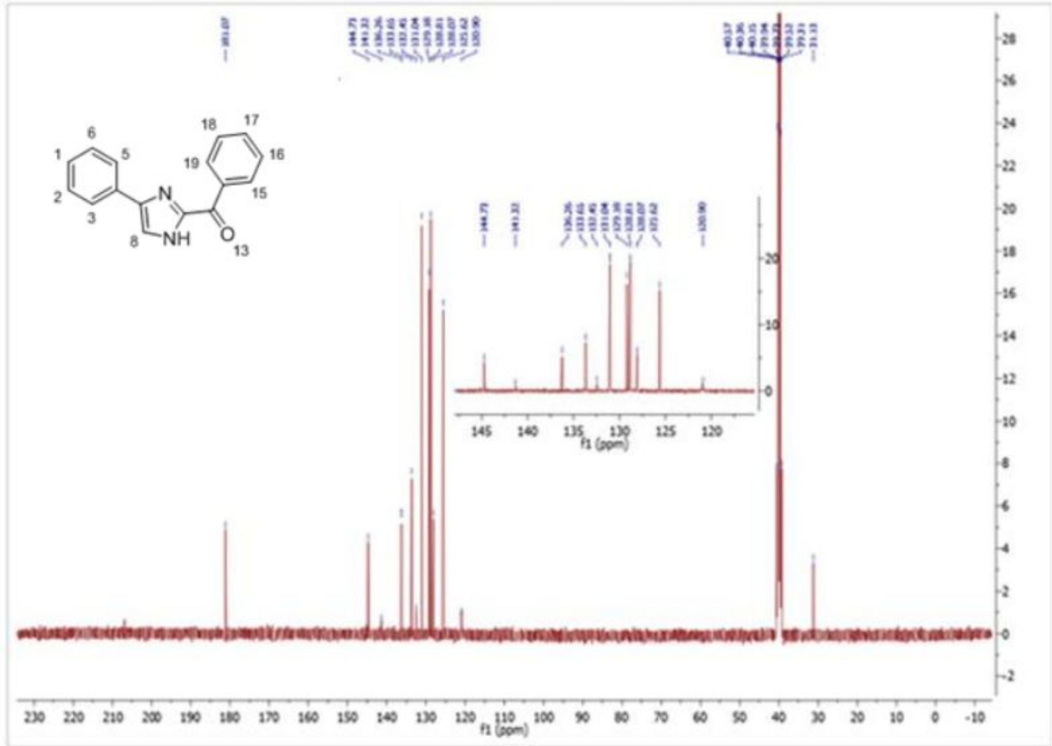

Figure S4. ^{13}C-NMR spectrum for compound 1a

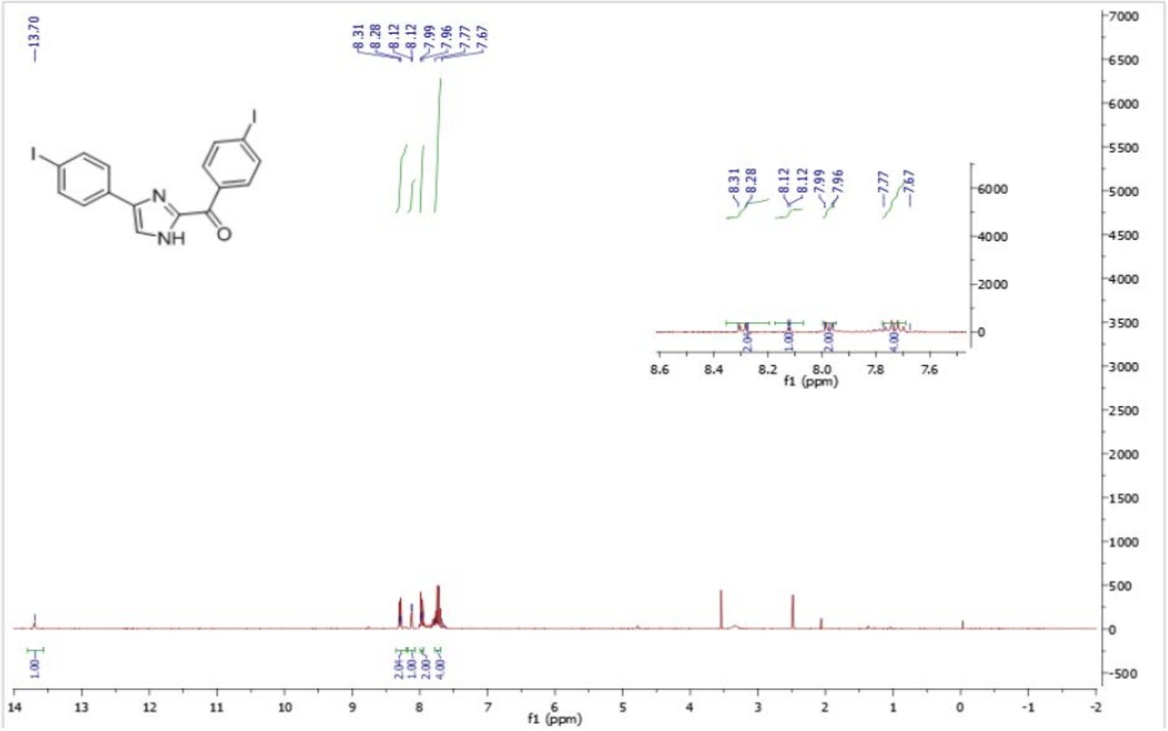

Figure S5. ^{1}H-NMR spectrum for compound 1b

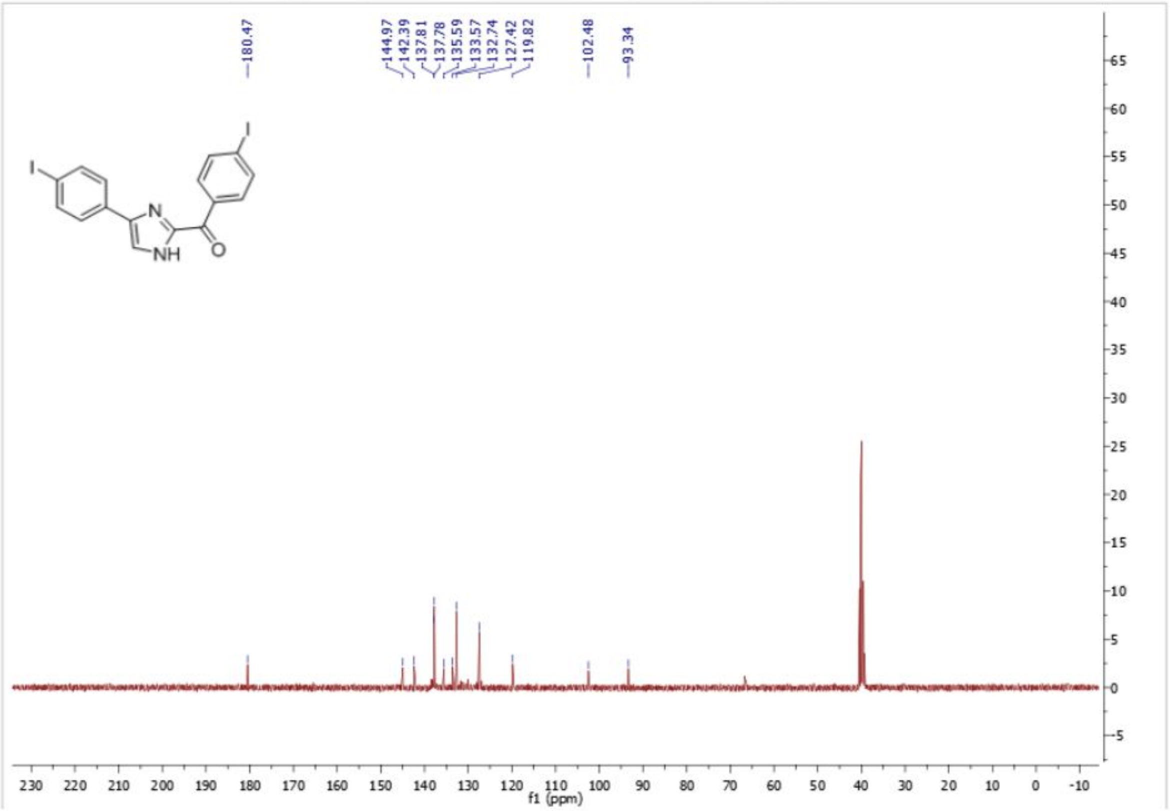

Figure S6. ^{13}C-NMR spectrum for compound 1b

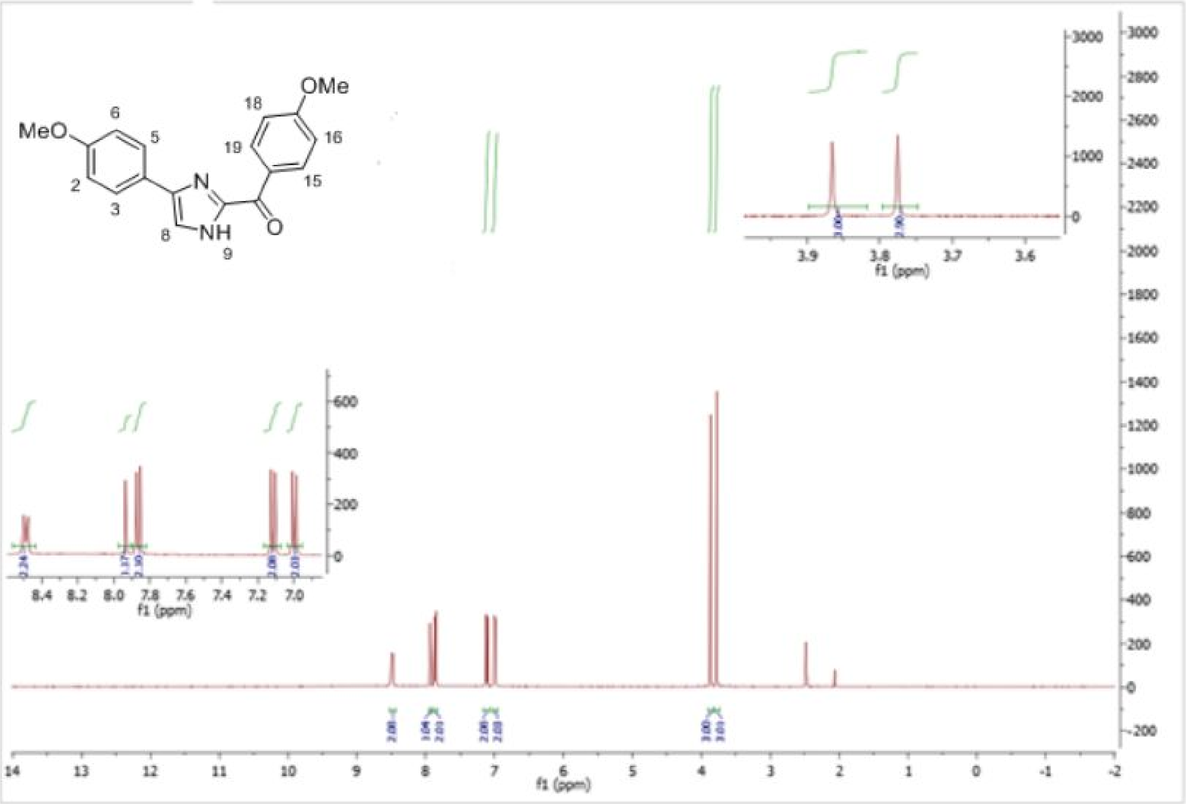

Figure S7. ^{1}H-NMR spectrum for compound 1c

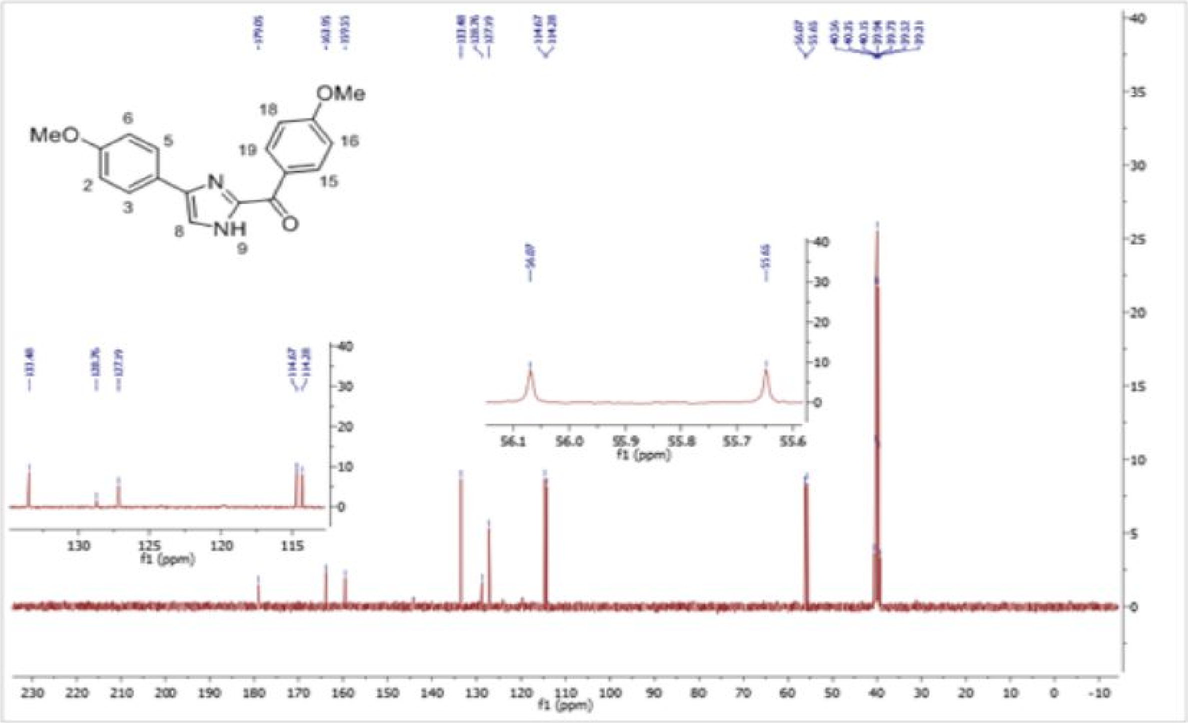

Figure S8. ^{13}C-NMR spectrum for compound 1c

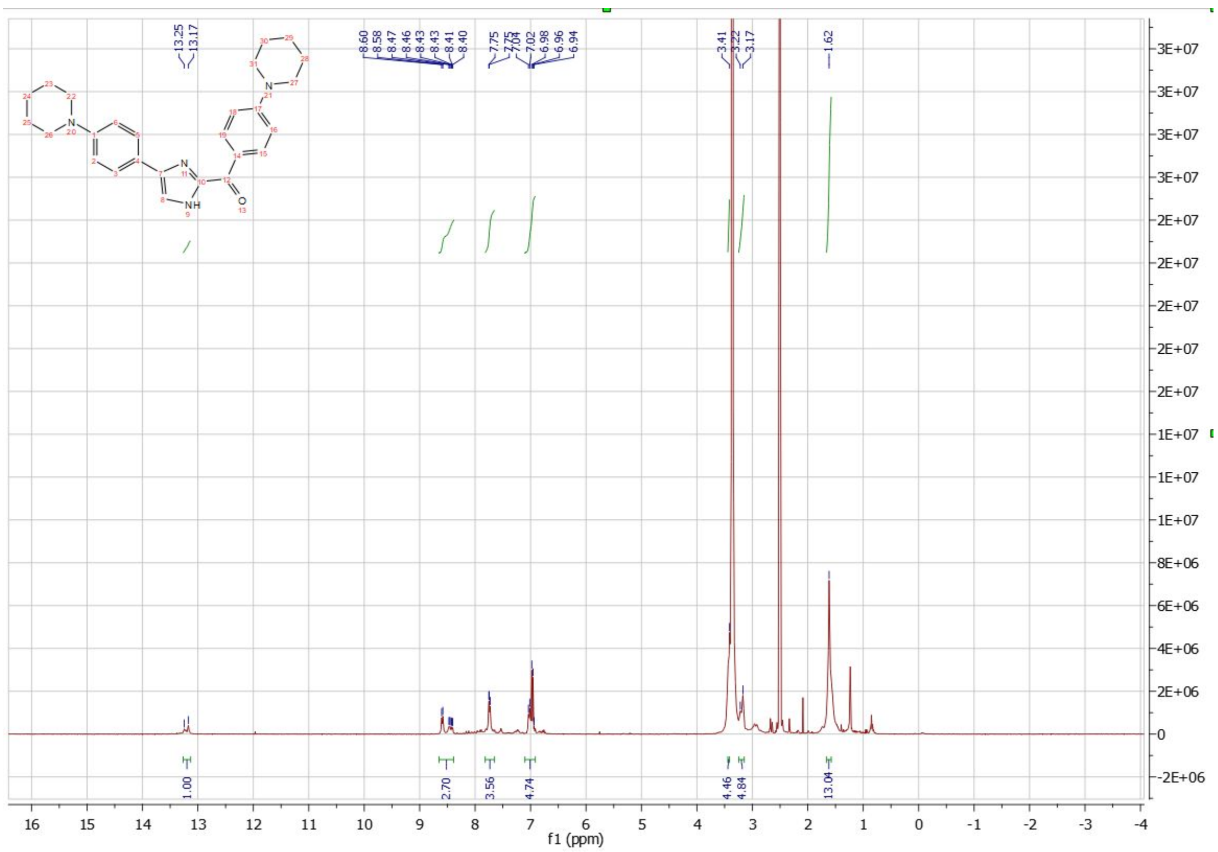

Figure S9. ^{1}H-NMR spectrum for compound 1d

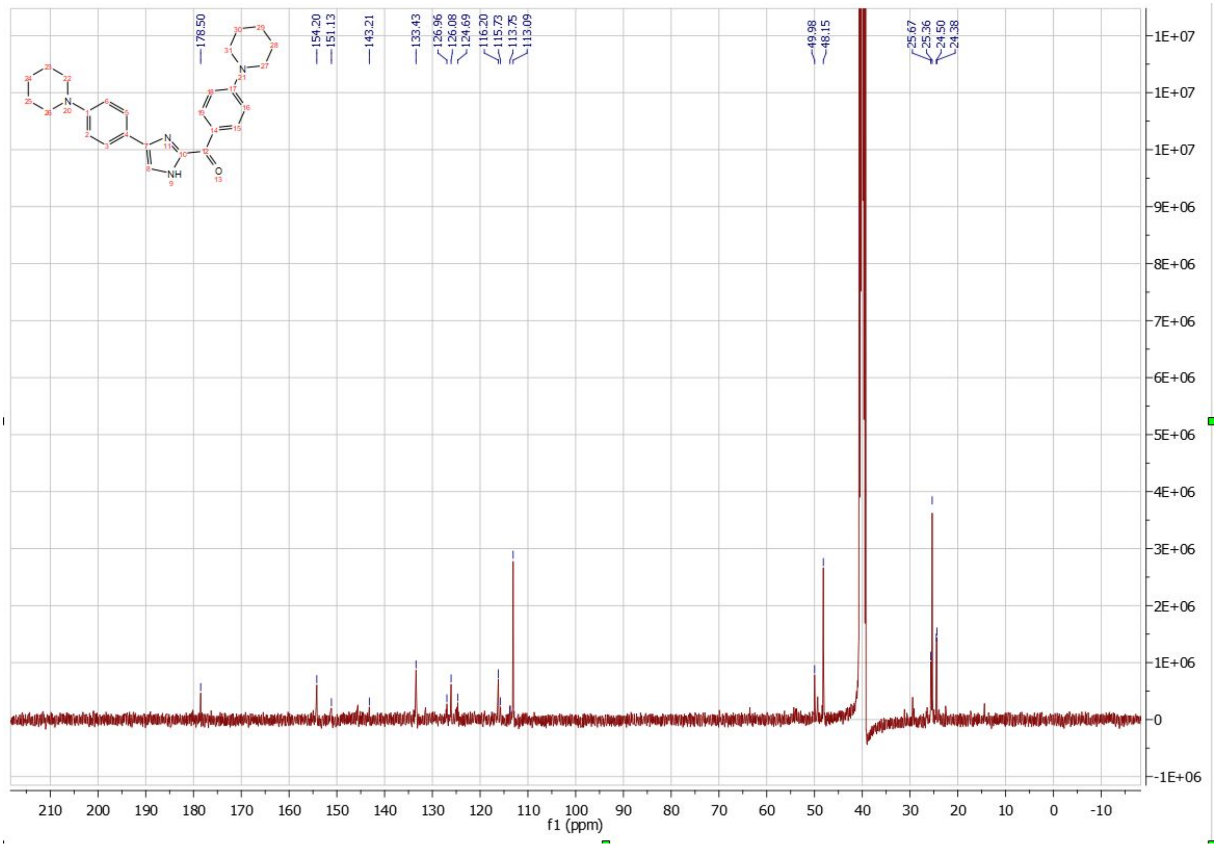

Figure S10. ^{13}C-NMR spectrum for compound 1d

Sample Information

Data Filename	: Sergen_4.lcd
Method Filename	: Büşra.lcm
Date Acquired	: 8.02.2024 11:54:19
Data Processed	: 8.02.2024 12:14:22

Chromatogram
Sergen_4 C:\Users\Hp\Desktop\Datalar\Büşra\Sergen_4.lcd

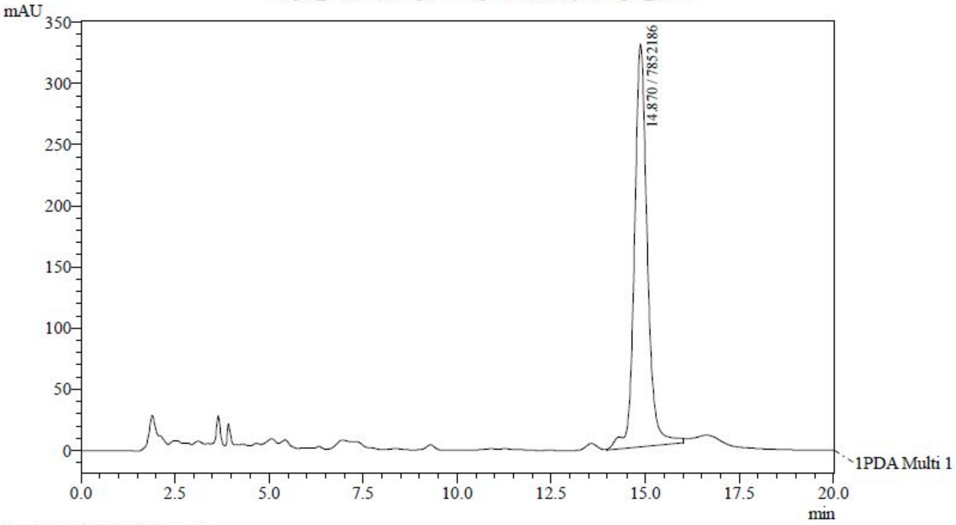

1 PDA Multi 1 / 360nm 4nm

PeakTable

Ch1 360nm 4nm

Peak#	Ret. Time	Area	Height	Area %
1	14.870	7852186	329229	100.000
Total		7852186	329229	100.000

Figure S11. HPLC spectrum for compound 1d
